# 3D-CT reconstruction for pedicle outer width assessment in patients with thoracolumbar spine fractures: a comparative analysis between age groups <60 years and ≥60 years

**DOI:** 10.3389/fsurg.2024.1407484

**Published:** 2024-07-03

**Authors:** Qiang He, Yifeng Yan, Jie Mei, Chengxin Xie, Xin Sun

**Affiliations:** ^1^ Department of Orthopedics, Nanjing Hospital of Traditional Chinese Medicine Affiliated to Nanjing University of Chinese Medicine, Nanjing, China; ^2^ Department of Orthopedics, Shandong University of Traditional Chinese Medicine Affiliated Hospital, Jinan, China; ^3^ Department of Orthopedics, Jiangsu Province Second Hospital of Traditional Chinese Medicine, Nanjing, China; ^4^ Faculty of Graduate Studies, Shandong First Medical University, Jinan, China; ^5^ Department of Orthopedics, Taizhou Hospital of Zhejiang Province Affiliated to Wenzhou Medical University, Taizhou, China

**Keywords:** three-dimensional CT reconstruction, thoracolumbar spine fracture, pedicle outer width, osteoporosis vertebral fractures, vertebral pedicle

## Abstract

**Objective:**

This study aims to compare the utilization of 3D-CT reconstruction in measuring pedicle outer width (POW) between younger/middle-aged patients (<60 years) and older patients (≥60 years) with thoracolumbar spine fractures (TSF).

**Methods:**

We conducted a retrospective study from January 2021 to December 2022, involving a total of 108 patients with TSF. The study population consisted of 62 patients aged ≥60 years (observation group) and 46 patients aged <60 years (control group). We compared the POW on both the right and left sides of the thoracolumbar spine between the two groups. Additionally, we analyzed the POW by gender within each group and calculated the incidence of patients falling below the critical values for arch root puncture (5 mm) and arch root nailing (7 mm) in both groups.

**Results:**

There were no statistically significant differences observed in the POW between the two groups on both the left and right sides of each corresponding vertebra (*P* > 0.05). In the observation group, both male and female patients had significantly smaller POW compared to the control group (*P* < 0.05). However, no significant difference in POW was observed between the same-sex groups in the L4 to L5 vertebrae (*P* > 0.05). In the observation group, the POW was less than 5 mm in 9.33% (81/868) of cases and less than 7 mm in 49.88% (433/868) of cases, primarily observed from T11 to L3. In the control group, 4.81% (31/644) of cases had a POW of less than 5 mm, and 13.81% (88/644) had a POW of less than 7 mm.

**Conclusion:**

Utilizing preoperative 3D-CT reconstruction to measure POW in patients with TSF not only facilitates the assessment of surgical feasibility but also aids in surgical pathway planning, thus potentially reducing the incidence of postoperative complications.

## 1 Introduction

Thoracolumbar spine fractures (TSF) are primarily caused by osteoporosis in elderly patients, often triggered by minor trauma. The severity of the disease can be exacerbated by significantly reduced bone strength and disrupted bone balance (1). With the society undergoing progressive aging, there has been a notable increase in the number of elderly patients seeking medical treatment. Surgical intervention currently remains the primary approach, with percutaneous kyphoplasty (PKP) being a commonly utilized procedure in clinical practice. PKP is renowned for its minimally invasive nature, effective pain relief, and ability to restore vertebral height, thereby serving as the cornerstone of surgical management for TSF (2, 3). However, the occurrence of postoperative complications, including pedicle wall fractures, spinal cord compression, and nerve root injuries, closely relates to the anatomical characteristics of the pedicle. Therefore, accurate measurement of pedicle morphology and dimensions becomes crucial (4).

This retrospective analysis comprises 108 TSF patients (T11 to L5) and aims to compare the changes and characteristics of POW measurements in two distinct age groups (age <60 years and ≥60 years), providing valuable insights for clinical surgical practice.

## 2 Methods

### 2.1 Study setting and subjects

This retrospective study utilized electronic medical records (EMR) from Nanjing Hospital of Traditional Chinese Medicine Affiliated to Nanjing University of Chinese Medicine to collect patient data who were received treatment between January 2021 and December 2022. Demographics data (i.e., age and sex), course of disease records, prescription drug dispensation records, bone mineral density (BMD) data, and fracture site records were captured.

The inclusion criteria were as follows: (1) age ≥ 18 years; (2) confirmed diagnosis of TSF, including osteoporotic vertebral compression fractures (OVCF) caused by minor trauma; (3) no history of spinal fractures before TSF; (4) a definite history of trauma. The exclusion criteria were as follows: (1) patients with vertebral tumors or tuberculosis; (2) patients with infectious diseases, coagulation disorders, or spinal cord nerve injuries; (3) patients with vertebral pedicle fractures or dislocations that hindered the measurement of POW; and (4) patients with poor adherence or who discontinued follow-up.

The study followed the Declaration of Helsinki (revised in 2013) and was approved by the ethics committee of Nanjing Hospital of Traditional Chinese Medicine Affiliated to Nanjing University of Chinese Medicine. All patients included in this study provided informed consent for the surgical protocol.

### 2.2 POW measurement

**Figure 1:**
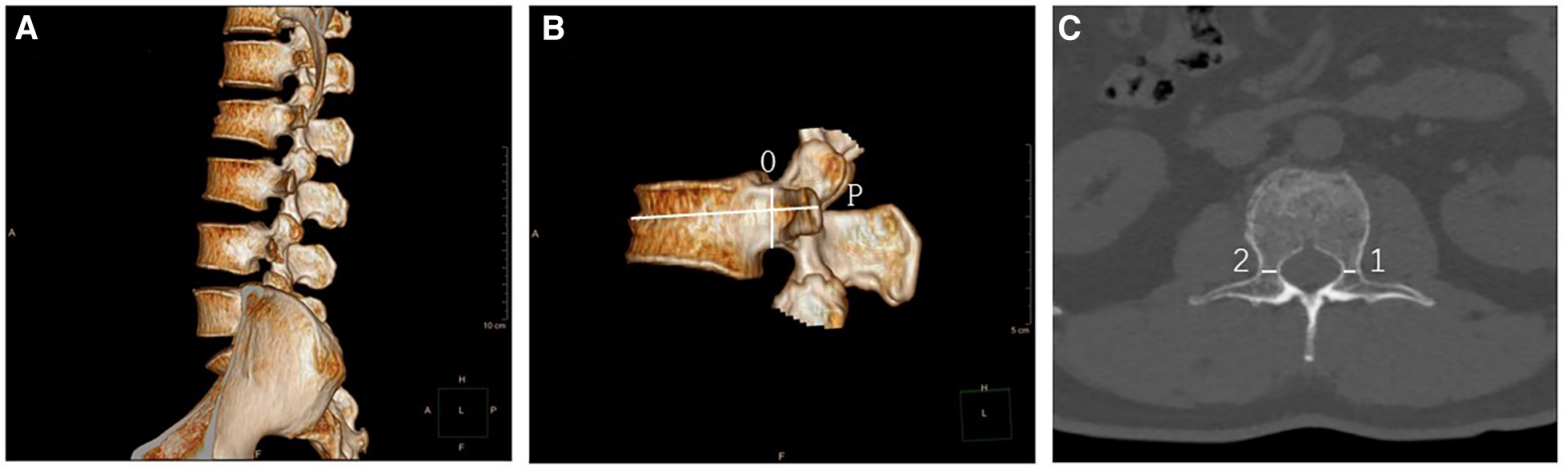
(**A**) A multidimensional image from T11 to L5, highlighting the significant wedge-shaped flattening of the L2 vertebral body. (**B**) A lateral image identifying the position of the pedicle axis (P). (**C**) A cross-sectional view used to measure the POW value.

The POW measurements of thoracolumbar spine (T11 to L5) were measured by Revolution 256-row CT machine (General Electric, USA) with a dose of 120 kV and 250 mA. The acquired images were transferred to the ADW4.6 workstation for processing and storage. The images had a layer thickness and layer spacing of 0.625 mm, a window width of 1,300 Hu, a window position of 400 Hu, and a distance accuracy of 0.1 mm. Surface-masked images of T11 to L5 were generated using techniques such as stage limitation and regional clipping. Reconstruction parameters were adjusted, while the soft tissues surrounding the vertebral body were shielded, resulting in the acquisition of multidimensional images (Figure 1A). The center of the shortest distance from the top and bottom walls of the pedicles was selected to be O, and the axis of the pedicle was drawn as *P* (Figure 1B). The POW was defined as the distance between the medial and lateral bone cortex at the narrowest point of the pedicle, passing through *P* and parallel to the cross-sectional image of the upper endplate (Figure 1C).

### 2.3 Outcome indicators

We measured the POW of the thoracolumbar spine (T11 to L5) on the left and right sides of the corresponding vertebrae in the patients. Subsequently, we conducted a comparison of the POW measurements between the two groups. Furthermore, we analyzed and compared the POW measurements of the thoracolumbar spine between the two groups across different genders. To determine the incidence of patients falling below the threshold values for pedicle impingement (POW < 5 mm) and pedicle implantation (POW < 7 mm), we referenced the threshold values used in both domestic and international clinical settings and calculated the measurements accordingly in the two groups.

### 2.4 Statistical methods

All analyses were conducted with SPSS (version 24.0, IBM, Inc., New York, USA). Continuous variables were calculated using a t-test and presented as the mean ± standard deviation (mean ± SD). Categorical variables were calculated using a chi-square test and presented as frequencies (%). *P* < 0.05 was considered significant statistically.

## 3 Results

### 3.1 Baseline characteristics

A total of 108 patients meets the inclusion and exclusion criteria were included in this study. The observation group consisted of 62 elderly patients (age ≥60 years). Among these, 48 patients had a single vertebral compression fracture, and 14 patients had two or more fractures. The control group consisted of 46 young and middle-aged patients (age <60 years). Among these, 38 patients had a single vertebral fracture, while 8 patients had two or more fractures. The baseline characteristics of patients see Table 1.

**Table 1:** Baseline characteristics of patients.

Groups	Age (years)	Sex	BMD *T*-score (mean ± SD)	Disease duration (days)	Fracture sites (cases)						
					T11	T12	L1	L2	L3	L4	L5
Observation group (*n* = 62)	73.25 ± 6.67	44F/18M	−3.32 ± 0.71	21.09 ± 7.27	6	12	32	21	9	3	1
Control group (*n* = 46)	38.91 ± 8.54	17F/29M	Not measured	38.91 ± 8.54	3	8	29	16	5	2	1
*t/x* ^2^	10.294	6.289		8.278	1.089						
*P*	<0.001	<0.001		<0.001	>0.05						

### 3.2 Comparison of POW between the left and right sides of each corresponding vertebra

There was no statistically significant difference (*P *> 0.05) in POW measurements between the left and right sides of each corresponding vertebra (T11 to L5) within both groups (Table 2). Therefore, the average of the POW measurements from the left and right sides of each corresponding vertebra was calculated and used as the POW value for the respective pedicle.

**Table 2:** Comparison of POW between the right and left sides of each corresponding vertebra (mean ± SD, mm).

Groups		T_11_	T_12_	L_1_	L_2_	L_3_	L_4_	L_5_
Observation group (*n* = 62)	Left side	6.20 ± 0.88	6.61 ± 0.96	5.61 ± 0.97	6.34 ± 0.91	7.31 ± 0.98	10.12 ± 1.58	12.46 ± 1.43
	Right side	6.29 ± 0.90	6.79 ± 0.97	5.71 ± 0.94	6.40 ± 0.92	7.36 ± 1.01	10.19 ± 1.54	12.58 ± 1.47
*T*		0.698	0.677	0.718	0.412	0.343	0.512	0.582
*P*		0.488	0.521	0.461	0.684	0.735	0.617	0.566
Control group (*n* = 46)	Left side	7.63 ± 1.16	8.22 ± 1.13	7.43 ± 1.17	7.83 ± 1.15	8.43 ± 1.12	10.49 ± 1.34	12.82 ± 1.14
	Right side	7.71 ± 1.41	8.29 ± 1.17	7.51 ± 1.22	7.92 ± 1.21	8.51 ± 1.18	10.54 ± 1.41	12.91 ± 1.23
*t*		0.724	0.511	0.691	0.688	1.218	0.917	0.617
*P*		0.425	0.621	0.476	0.491	0.114	0.282	0.502

### 3.3 Comparison of POW between the two groups

As shown in Table 3, in the observation group, the POW measurements of each corresponding vertebra from T11 to L3 were found to be smaller compared to those in the control group (*P* < 0.05). However, there was no statistically significant difference in POW measurements of L4 to L5 between the two groups (*P *> 0.05).

**Table 3:** Comparison of POW between the two groups (mean ± SD, mm).

Groups		T_11_	T_12_	L_1_	L_2_	L_3_	L_4_	L_5_
Observation group (*n* = 62)	Realm	4.21∼9.03	4.14∼9.45	3.45∼8.66	4.42∼9.36	4.82∼10.48	6.12∼14.18	8.56∼16.25
	Average value	6.25 ± 0.88*	6.65 ± 0.98*	6.18 ± 1.31*	6.36 ± 0.93*	7.33 ± 1.01*	10.13 ± 1.52	12.53 ± 1.44
Control Group (*n* = 46)	Realm	5.11∼9.98	4.88∼10.70	4.47∼10.24	5.02∼10.88	5.67∼11.38	7.02∼13.94	9.77∼15.89
	Average value	7.66 ± 1.08	8.26 ± 1.18	7.46 ± 1.19	7.87 ± 1.22	8.46 ± 1.18	10.31 ± 1.36	12.84 ± 1.21
*t*		0.724	0.511	0.691	0.688	1.218	0.917	0.617
*P*		0.425	0.621	0.476	0.491	0.114	0.282	0.502

Compared with the control group.

* *P *< 0.05.

### 3.4 Comparison of POW between the genders

In both the observation and control groups, the POW measurements of male patients from T_11_ to L_3_ were found to be greater than those of female patients (*P *< 0.05). However, there was no significant difference in the POW of L_4_ to L_5_ between males and females in two groups (*P *> 0.05). In the comparison of POW within the same gender between the two groups, the POW measurements in each corresponding vertebra of the T12 to L3 were smaller in the observation group compared to the control group (*P* < 0.05). However, there was no significant difference in the POW of L4 to L5 within the same gender between the two groups. (*P *> 0.05). See Table 4.

**Table 4:** Comparison of POW between the genders (mean ± SD, mm).

Groups		T_11_	T_12_	L_1_	L_2_	L_3_	L_4_	L_5_
Male	Observation group (*n* = 62)	6.65 ± 0.96*	7.14 ± 1.11*	6.41 ± 1.14*	6.79 ± 1.01*	7.77 ± 1.13*	11.53 ± 1.36	13.62 ± 1.41
	Control group (*n* = 46)	7.81 ± 1.13**	8.38 ± 1.47**	7.57 ± 1.21**	8.07 ± 1.22**	8.66 ± 1.20**	11.01 ± 1.22	13.08 ± 1.26
*t*		0.698	10.152	11.718	9.296	16.916	4.683	0.916
*P*		0.488	<0.001	<0.001	0.003	<0.001	0.033	0.163
Female	Observation group (*n* = 62)	6.09 ± 0.82	6.46 ± 0.83	6.11 ± 1.39	6.22 ± 0.88	7.21 ± 0.93	9.63 ± 1.26	12.12 ± 1.25
	Control group (*n* = 46)	7.45 ± 1.07	8.08 ± 1.16	7.27 ± 1.10	7.62 ± 1.09	8.18 ± 1.17	9.71 ± 1.19	12.45 ± 1.12
*t*		0.724	9.075	15.518	10.926	11.064	7.298	0.728
*P*		0.425	0.006	<0.001	<0.001	<0.001	0.015	0.342

Compared with the female in observation group.

* *P *< 0.05; Compared with the female in control group, **P < 0.05.

### 3.5 Occurrence of below the threshold for pedicle puncture and nail placement

In the observation group, a total of 868 pedicles were measured, and 9.33% (81/868) of them had a POW measurement below the critical value for pedicle puncture (<5 mm). The POW below the critical value for pedicle implantation (<7 mm) accounting for 49.88% (433/868), with the majority of these measurements observed from T11 to L3. In the control group, a total of 644 pedicles were measured. Among them, 4.81% (31/644) had a POW measurement below 5 mm and 13.66% (88/644) had a POW measurement below 7 mm. These measurements were primarily distributed from T11 to L3. See Table 5.

**Table 5:** Occurrence of POW below the threshold for pedicle puncture and nail placement.

	Observation group males(*n* = 36)		Observation group females (*n* = 88)		Control group males (*n* = 58)		Control group females (*n* = 34)	
	<5 mm	<7 mm	<5 mm	<7 mm	<5 mm	<7 mm	<5 mm	<7 mm
T_11_	1 (2.78)	16 (44.44)	16 (18.18)	81 (92.05)	0 (0)	11 (18.97)	7 (20.59)	12 (35.29)
T_12_	1 (2.78)	12 (33.33)	12 (13.64)	74 (84.09)	0 (0)	8 (13.79)	3 (8.82)	4 (11.76)
L_1_	6 (16.67)	28 (77.78)	22 (25.00)	84 (95.45)	5 (8.62)	14 (24.14)	9 (26.47)	11 (32.35)
L_2_	2 (5.56)	13 (36.11)	14 (15.90)	73 (82.95)	0 (0)	9 (15.52)	5 (14.71)	9 (26.47)
L_3_	1 (2.78)	4 (11.11)	6 (6.82)	41 (46.59)	0 (0)	5 (8.62)	2 (5.88)	5 (14.71)
L_4_	0 (0)	0 (0)	0 (0)	7 (7.95)	0 (0)	0 (0)	0 (0)	0 (0)
L_5_	0 (0)	0 (0)	0 (0)	0 (0)	0 (0)	0 (0)	0 (0)	0 (0)
total	11 (4.37)	73 (28.97)	70 (11.36)	360(58.44)	5(1.23)	47(11.58)	26(10.92)	41(17.23)

[*n* (%)].

## 4 Discussion

TSF is a prevalent type of fracture observed in clinical spinal surgery, particularly among the elderly population (5, 6). It is often attributed to factors such as gastrointestinal dysfunction, impaired absorption of calcium, decreased bone formation, mineralization capacity, and reduced BMD. With the bone trabeculae becoming less dense and the bones becoming more brittle, TSF can occur even in the absence of apparent causal factors or with minimal external force exerted (7, 8).

The diameter of the vertebral pedicle gradually widens with age within certain age brackets, indicating a continuous alteration. Specifically, it widens progressively in adulthood, with females ceasing to show increases after the age of 50 and males after 60, thereafter exhibiting a diminishing trend (9, 10, 11). Our study findings revealed that older patients with TSF had smaller vertebral POW measurements compared to young and middle-aged individuals, specifically in the range from T11 to L3 (*P* < 0.05). In addition, our investigation revealed a gender disparity in the POW measurements of the thoracolumbar vertebrae (T11 to L3) within the same cohort. Specifically, males have exhibited larger POW measurements in contrast to females. Notably, there was a male-to-female ratio of 9:22 among elderly patients, indicating that female patients were more susceptible to TSF.

The strength of the lumbar extensor muscles decreases with age, this gradual weakening contributes to the development of stress changes in the spine, particularly affecting the vulnerability of the anterior spine to osteoporotic vertebral compression fractures. In addition, the age-related decline in the strength of the lumbar extensors leads to alterations in spinal stress distribution, further results in increased pressure on the anterior column of the spine, increased angle of thoracic kyphosis, decreased angle of lumbar lordosis, and a shift in the body's center of gravity. Consequently, these changes contribute to remodeling of the vertebral arches. It is noteworthy that while the T11 and T12 vertebrae are still connected to the ribs, they do not significantly contribute to the formation of the thoracic contour. Therefore, the stress concentration in the spinal region shifts from the thoracic to the lumbar anterior convexity. As a result, TSF most commonly occurs between the T11 vertebra and the L3 vertebra, with a particularly high prevalence at the L1 and L2 vertebrae. Furthermore, the significant hormonal changes that occur in elderly female patients after menopause make them more susceptible to osteoporosis, increasing their risk of fractures.

Currently, surgical treatment remains the preferred approach for achieving efficient recovery in patients with TSF. In particular, the pedicle plays an indispensable role in PKP, which is a commonly employed surgical procedure for treating TSF (12). The assessment of pedicle parameters, particularly POW, is crucial for the successful execution of surgical procedures. The reduction in POW significantly impacts intraoperative vertebral pedicle puncture procedures. A POW of less than 5 mm indicates a narrow vertebral pedicle, making it unsuitable for using standard-sized puncture catheters (13). As POW decreases, there arises a necessity to adjust the catheter diameter. Therefore, preoperative POW measurements can provide direct evidence for selecting the appropriate puncture catheter during the procedure. In addition, in patients presenting with severe spinal instability, spinal cord injury, spinal tumors, and similar conditions, vertebral pedicle screw insertion procedures are warranted (14). A diminutive POW may exacerbate the difficulty of screw insertion, potentially leading to complications such as fractures of the inner and outer walls of the pedicle (14).Therefore, when performing vertebral pedicle screw insertion procedures, it is imperative to calculate the appropriate critical value for pedicle screw placement based on preoperative POW measurements, aiming to mitigate postoperative complications.

POW, as one of the crucial parameters, serves as a valuable tool for clinicians to discern the anatomical characteristics of the vertebral pedicle (15). The critical values for pedicle puncture (POW < 5 mm) and pedicle nail placement (POW < 7 mm) have been established (16). When the POW falls below these critical values, it is not advisable to utilize conventional puncture instruments for the operation. Therefore, precise determination of the POW value is essential for procedural success.

## 5 Conclusion

In this study, we observed that the percentage of patients with POW measurements below the critical value for pedicle puncture (5 mm) and the critical value for pedicle nail placement (7 mm) in the observation group was higher than that of control group. In addition, in the observation group and the control group, the percentage of females with POW below 5 mm was higher than the males in the same group. The percentage of females with POW measurements below 7 mm was higher than the males in the same group. These findings indicate the importance of exercising additional caution when performing pedicle puncture, particularly in females, especially when the fracture involves the vertebral levels ranging from the T12 to L2 vertebrae.

## Data Availability

The original contributions presented in the study are included in the article/Supplementary Material, further inquiries can be directed to the corresponding authors.
